# MYC/MIZ1-dependent gene repression inversely coordinates the circadian clock with cell cycle and proliferation

**DOI:** 10.1038/ncomms11807

**Published:** 2016-06-24

**Authors:** Anton Shostak, Bianca Ruppert, Nati Ha, Philipp Bruns, Umut H. Toprak, ICGC MMML-Seq Project, Chris Lawerenz, Peter Lichter, Bernhard Radlwimmer, Jürgen Eils, Benedikt Brors, Sylwester Radomski, Ingrid Scholz, Gesine Richter, Reiner Siebert, Susanne Wagner, Andrea Haake, Julia Richter, Sietse Aukema, Ole Ammerpohl, Christina Lopez, Inga Nagel, Inga Vater, Rabea Wagner, Christoph Borst, Siegfried Haas, Marius Rohde, Birgit Burkhardt, Jasmin Lisfeld, Alexander Claviez, Martin Dreyling, Sonja Eberth, Lorenz Trümper, Dieter Kube, Christina Stadler, Hermann Einsele, Norbert Frickhofen, Martin-Leo Hansmann, Dennis Karsch, Michael Kneba, Luisa Mantovani-Löffler, Peter Staib, Stephan Stilgenbauer, German Ott, Ralf Küppers, Marc Weniger, Michael Hummel, Dido Lenze, Monika Szczepanowski, Wolfram Klapper, Ulrike Kostezka, Peter Möller, Andreas Rosenwald, Ellen Leich, Jordan Pischimariov, Vera Binder, Arndt Borkhardt, Kebria Hezaveh, Jessica Hoell, Philip Rosenstiel, Markus Schilhabel, Stefan Schreiber, Stephan H. Bernhart, Gero Doose, Steve Hoffmann, Helene Kretzmer, David Langenberger, Hans Binder, Lydia Hopp, Markus Kreuz, Markus Loeffler, Maciej Rosolowski, Jan Korbel, Stefanie Sungalee, Peter F. Stadler, Thorsten Zenz, Roland Eils, Matthias Schlesner, Axel Diernfellner, Michael Brunner

**Affiliations:** 1 Heidelberg University, Biochemistry Center, Im Neuenheimer Feld 328, D-69120 Heidelberg, Germany; 2 Division Theoretical Bioinformatics (B080), German Cancer Research Center (DKFZ), Im Neuenheimer Feld 280, D-69120 Heidelberg, Germany; 3 Department for Bioinformatics and Functional Genomics, Institute for Pharmacy and Molecular Biotechnology (IPMB) and BioQuant, Heidelberg University, Im Neuenheimer Feld 364, D-69120 Heidelberg, Germany; 4 Institute of Human Genetics, University Hospital Schleswig-Holstein Campus Kiel/Christian-Albrechts University Kiel, Kiel D-24105, Germany; 5 Friedrich-Ebert Hospital Neumünster, Clinics for Hematology, Oncology and Nephrology, Neumünster D-24534, Germany; 6 Department of Pediatric Hematology and Oncology, University Hospital Münster, Münster D-48149, Germany; 7 Department of Pediatric Hematology and Oncology, University Hospital Giessen, Giessen D-35043, Germany; 8 Department of Pediatrics, University Hospital Schleswig-Holstein, Campus Kiel, Kiel D-24105, Germany; 9 Department of Medicine III - Campus Grosshadern, University Hospital Munich, Munich D-81377, Germany; 10 Department of Hematology and Oncology, Georg-August-University of Göttingen, Göttingen D-37075, Germany; 11 University Hospital Würzburg, Department of Medicine and Poliklinik II, University of Würzburg, Würzburg D-97080, Germany; 12 Department of Medicine III, Hematology and Oncology, Dr. Horst-Schmidt-Kliniken of Wiesbaden, Wiesbaden D-65199, Germany; 13 Senckenberg Institute of Pathology, University of Frankfurt Medical School, Frankfurt am Main D-60590, Germany; 14 Department of Internal Medicine II: Hematology and Oncology, University Medical Centre, Campus Kiel, Kiel D-24105, Germany; 15 Hospital of Internal Medicine II, Hematology and Oncology, St-Georg Hospital Leipzig, Leipzig D-04129, Germany; 16 Department of Oncology, Hematology and stem cell transplantation, Univesity Hospital Aachen, St.Antonius Hospital, University of Aachen, Aachen D-52074, Germany; 17 Department of Internal Medicine III, University of Ulm, Ulm D-89081, Germany; 18 Department of Pathology, Robert-Bosch Hospital Stuttgart, Stuttgart D-70376, Germany; 19 Institute of Cell Biology (Cancer Research), University of Duisburg-Essen, Essen D-45122, Germany; 20 Institute of Pathology, Charité – University Medicine Berlin, Berlin D-10117, Germany; 21 Hematopathology Section, University Hospital Schleswig-Holstein Campus Kiel/Christian-Albrechts University Kiel, Kiel D-24105, Germany; 22 Comprehensive Cancer Center Ulm (CCCU), University Hospital Ulm, Ulm D-89081, Germany; 23 Institute of Pathology, Medical Faculty of the Ulm University, Ulm D-89081, Germany; 24 Institute of Pathology, University of Würzburg, Würzburg D-97080, Germany; 25 Department of Pediatric Oncology, Hematology and Clinical Immunology, Heinrich-Heine-University, Düsseldorf D-40225, Germany; 26 Institute of Clinical Molecular Biology, University Hospital Schleswig-Holstein Campus Kiel/Christian-Albrechts University Kiel, Kiel D-24105, Germany; 27 Department of General Internal Medicine, University Hospital Schleswig-Holstein Campus Kiel/Christian-Albrechts University Kiel, Kiel D-24105, Germany; 28 Transcriptome Bioinformatics Group, LIFE Research Center for Civilization Diseases, Leipzig D-04103, Germany; 29 Interdisciplinary Center for Bioinformatics, University of Leipzig, Leipzig D-04107, Germany; 30 Bioinformatics Group, Department of Computer Science, University of Leipzig, Leipzig D-04103, Germany; 31 Institute for Medical Informatics Statistics and Epidemiology, Leipzig D-04107, Germany; 32 EMBL Heidelberg, Genome Biology, Heidelberg D-69117, Germany; 33 RNomics Group, Fraunhofer Institute for Cell Therapy and Immunology IZI, Leipzig D-04103, Germany; 34 Santa Fe Institute, Santa Fe,New Mexico NM 87501, USA; 35 Max-Planck-Institute for Mathematics in Sciences, Leipzig D-04103, Germany; 36 Department of Medicine V, University of Heidelberg, Heidelberg D-69120, Germany

## Abstract

The circadian clock and the cell cycle are major cellular systems that organize global physiology in temporal fashion. It seems conceivable that the potentially conflicting programs are coordinated. We show here that overexpression of *MYC* in U2OS cells attenuates the clock and conversely promotes cell proliferation while downregulation of *MYC* strengthens the clock and reduces proliferation. Inhibition of the circadian clock is crucially dependent on the formation of repressive complexes of MYC with MIZ1 and subsequent downregulation of the core clock genes *BMAL1* (*ARNTL*), *CLOCK* and *NPAS2*. We show furthermore that *BMAL1* expression levels correlate inversely with *MYC* levels in 102 human lymphomas. Our data suggest that MYC acts as a master coordinator that inversely modulates the impact of cell cycle and circadian clock on gene expression.

## Abstract (web summary)

The circadian clock and the cell cycle systems coordinate global physiology. Here the authors show that MYC represses the clock genes, together with MIZ1, and induces proliferation, suggesting that MYC inversely modulates cell cycle and circadian clock genes.

## Graphical abstract

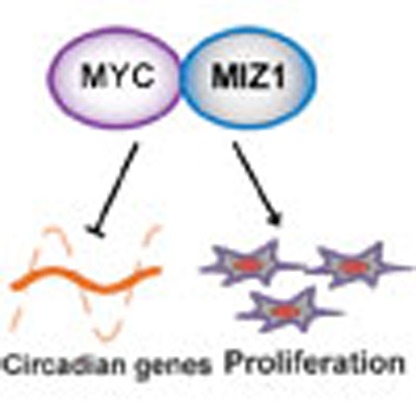

Many aspects of mammalian physiology and behaviour are rhythmically regulated by the circadian clock^1^. On a cellular level, the circadian clock is dependent on interconnected transcriptional/translational feedback loops. In brief, the core transcription activator complex BMAL1/CLOCK (or its homologue BMAL1/NPAS2) rhythmically activates expression of clock genes including *CRYs*, *PERs*, *REV-ERBs* and *RORs*. CRYs and PERs are inhibitors of CLOCK/BMAL, whereas REV-ERBs are repressors that control in coordination with ROR activators expression of *BMAL1*, *CLOCK* and *NPAS2*. The D-box-specific transcription factors E4BP, DBP, TEF and HLF additionally contribute to the regulation of specific clock genes^2^.

Disruption or misalignment of circadian rhythms in humans has been associated with numerous pathological conditions including cancer^3^,^4^. Mice with chronic jet-lag or disrupted circadian physiology due to lesions of their suprachiasmatic nuclei exhibit accelerated growth of tumours^5^,^6^. Vice versa, cancer types correlating with impaired cell cycle and proliferation frequently exhibit aberrant expression of clock genes^7^,^8^. *MYC* is an oncogene, which is found to be deregulated in different cancers and, amplification of MYC often correlates with tumour aggression and poor prognosis^9^. MYC and its partner MAX are, like the circadian transcription factors BMAL1, CLOCK and, NPAS2, members of the bHLH transcription factors family, which form heterodimers that bind to so-called E-box motifs. MYC regulates transcription of up to 15% of the transcriptome including genes involved in apoptosis, cell growth and proliferation^10^,^11^. Recently, MYC has been suggested to attenuate the circadian clock by activating via circadian E-box sites transcription and expression of REV-ERBα/β, which would then repress transcription of *BMAL1* (ref. 12). Since the DNA-binding specificity of MYC/MAX and CLOCK/BMAL1 complexes is highly similar, it seems conceivable that overexpressed MYC could constitutively activate and overexpress the E-box-dependent circadian repressor genes *REV-ERBα/β*, *PER1/2* and *CRY1/2*.

Here we show, that overexpression of MYC attenuates the circadian clock of U2OS cells. Downregulation of the clock by overexpressed MYC is dependent on MYC/MIZ1 complexes, which are recruited to non-E-box sites in the promoters of *BMAL1* and *CLOCK*. MYC/MIZ1 complexes stimulate proliferation of U2OS cells, suggesting that MYC inversely correlates the circadian clock and the cell cycle.

## Results

### BMAL1 and MYC share common target genes

**Figure 1:**
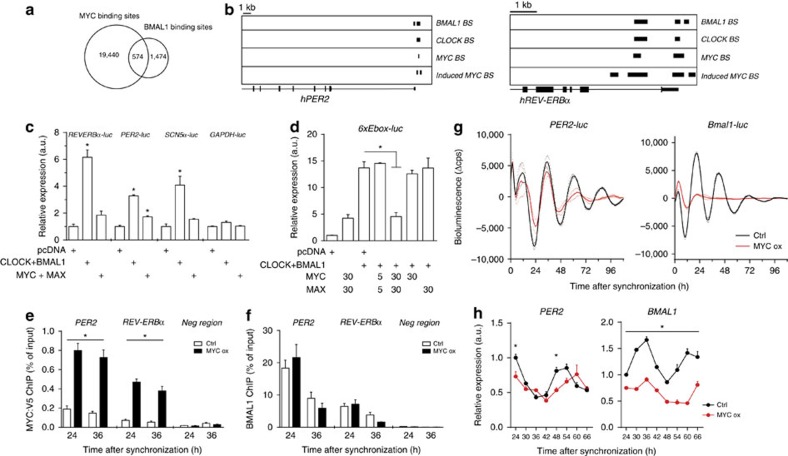
Overexpression of MYC attenuates the circadian clock. (**a**) Overlap between native MYC (ref. 14) and BMAL1 (ref. 13) binding sites in U2OS cells. (**b**) *PER2* and *REV-ERBα* loci with binding sites (BS) of BMAL1, CLOCK, native MYC and overexpressed MYC in U2OS cells (based on the data from refs 13, 14). (**c**) MYC and MAX do not substantially induce the BMAL1/CLOCK target genes *REV-ERBα, PER2* and *SCN5α*. HEK293 cells were transfected with *MYC*, *MAX*, *BMAL1* and *CLOCK* encoding plasmids (30 ng) together with the indicated circadian promoter-luc reporter plasmids. *GAPDH-luc* was transfected as a negative control (*n*=3). (**d**) MYC/MAX restricts stronger induction of *6xEbox-luc* by CLOCK/BMAL1. HEK293 cells were transfected with 30 ng of each *BMAL1* and *CLOCK* plasmids, and with the indicated amounts (in ng) of *MYC* and *MAX* vectors (*n*=3). ChIP-PCR analysis of (**e**) MYC:V5 and (**f**) BMAL1 binding to circadian E-boxes in *PER2* and *REV-ERBα* promoters in synchronized and doxycycline-induced U2OS *t-rex tetO-MYC:V5* cells (*n*=3). (**g**) Bioluminescence recorded from synchronized *Bmal1-luc* and *PER2-luc* U2OS *t-rex tetO-MYC:V5* cells (*n*=3). (**h**) Quantitative PCR (qPCR) analysis of circadian expression profiles of *PER2* and *BMAL1* transcripts in synchronized U2OS *t-rex tetO-MYC:V5* cells (*n*=3). Data are presented as mean±s.e.m. *P<0.05; one-way (**c**,**d**) and two-way (**e**,**h**) analysis of variance (ANOVA) with Bonferroni post-test.

We therefore tested whether and how MYC/MAX and CLOCK/BMAL1 might regulate common circadian genes. A comparison of published chromatin immunoprecipitation sequencing (ChIP-seq) data sets of U2OS cells revealed a significant overlap between both cistromes, with 28% (574/2048) of BMAL1 binding sites overlapping with sites also bound by MYC (refs 13, 14; Fig. 1a). Such common binding sites include the core circadian clock genes *REV-ERBα, PER1/2* and *DBP* as well as clock-controlled genes such as *SCN5A* (Fig. 1b and Supplementary Fig. 1a). However, co-transfection of HEK293 cells with MYC/MAX expressing constructs did, in contrast to CLOCK/BMAL1, not strongly activate the circadian reporter genes *REVERBα-luc, PER2-luc* and *SCN5A-luc* (Fig. 1c). To compare the activating potential of MYC/MAX and CLOCK/BMAL1 at E-boxes we assayed expression of a minimal promoter fused to 6 synthetic E-box elements (*6xEbox-luc*). Similar synthetic E-box reporters have been shown to be activated by MYC (ref. 15). Co-transfection of the *6xEbox-luc* reporter with *CLOCK* and *BMAL1* vectors resulted in notably higher luciferase activity than co-transfection with *MYC* and *MAX* vectors (14 fold versus 3–4 fold; Fig. 1d). Interestingly, simultaneous expression of MYC/MAX together with CLOCK/BMAL1 hampered activation of the *6xEbox-luc* reporter (Fig. 1d). Similarly, MYC/MAX interfered with stronger activation of *REVERBα-luc* and *PER2-luc* reporter genes by CLOCK/BMAL1 (Supplementary Fig. 1b). The data suggest that MYC/MAX has a weaker activation potential than CLOCK/BMAL1 at synthetic as well as endogenous circadian promoters. Yet, MYC/MAX is functionally dominant over CLOCK/BMAL1.

### Overexpression of MYC disrupts the circadian clock

Next, we generated a U2OS cell line expressing a doxycycline-inducible V5-tagged MYC (U2OS *t-rex tetO-MYC:V5*). In presence of doxycycline total MYC (endogenous+induced) was ∼9-fold overexpressed (Fig. 4d and Supplementary Fig. 4c).

Twenty four and thirty six hours after induction, MYC:V5 was efficiently recruited to circadian E-box sites in *REV-ERBα* and *PER2* (Fig. 1e). Rhythmic recruitment of BMAL1 to these loci was not compromised, yet BMAL1 occupancy was reduced 36 h after induction of MYC:V5 (Fig. 1f). The data suggest that at any given time the saturation level of the E-boxes with either transcription factor was rather low such that the transcription factors did not physically compete for common binding sites. The functional dominance of MYC/MAX could reflect a MYC/MAX induced chromatin state that allows binding of CLOCK/BMAL1 but interferes with stronger activation of target genes.

We then asked whether overexpression of MYC affects expression levels and circadian rhythms of clock genes. Induction of transgenic MYC:V5 attenuated the circadian expression rhythms of *PER2-luc* and *Bmal1-luc* reporters in synchronized U2OS cells, while expression of green fluorescent protein (control) had no effect (Fig. 1g and Supplementary Fig. 1c,d). Unexpectedly, however, the expression level and rhythm of the non-E-box-dependent *Bmal1-luc* reporter were strongly attenuated already shortly after induction of MYC:V5, whereas rhythmic expression of the E-box regulated *PER2-luc* reporter was affected with delayed kinetics (Fig. 1g). Surprisingly, expression levels of *PER2-luc* decreased in the presence of overexpressed MYC (Supplementary Fig. 1c) indicating that the MYC:V5 did not activate the E-box containing circadian promoter. Overexpression of MYC:V5 attenuated expression of endogenous *BMAL1* and blunted its circadian profile about 1 day earlier and more strongly than the rhythm of the E-box containing *PER2* gene (Fig. 1g,h). Furthermore, MYC:V5 expression caused downregulation of the non-E-box genes *CLOCK* and *NPAS2* (Supplementary Fig. 1e).

It has been suggested that MYC activates *REV-ERBα* via E-boxes, which in turn would downregulate *BMAL1* and thereby attenuate the circadian clock^12^. Indeed, overexpression of a doxycycline-inducible FLAG-tagged REV-ERBα repressed *Bmal1-luc* levels and damped its rhythms (Supplementary Fig. 2a), indicating that REV-ERBα is a potent repressor of *BMAL1* in U2OS cells.

**Figure 2:**
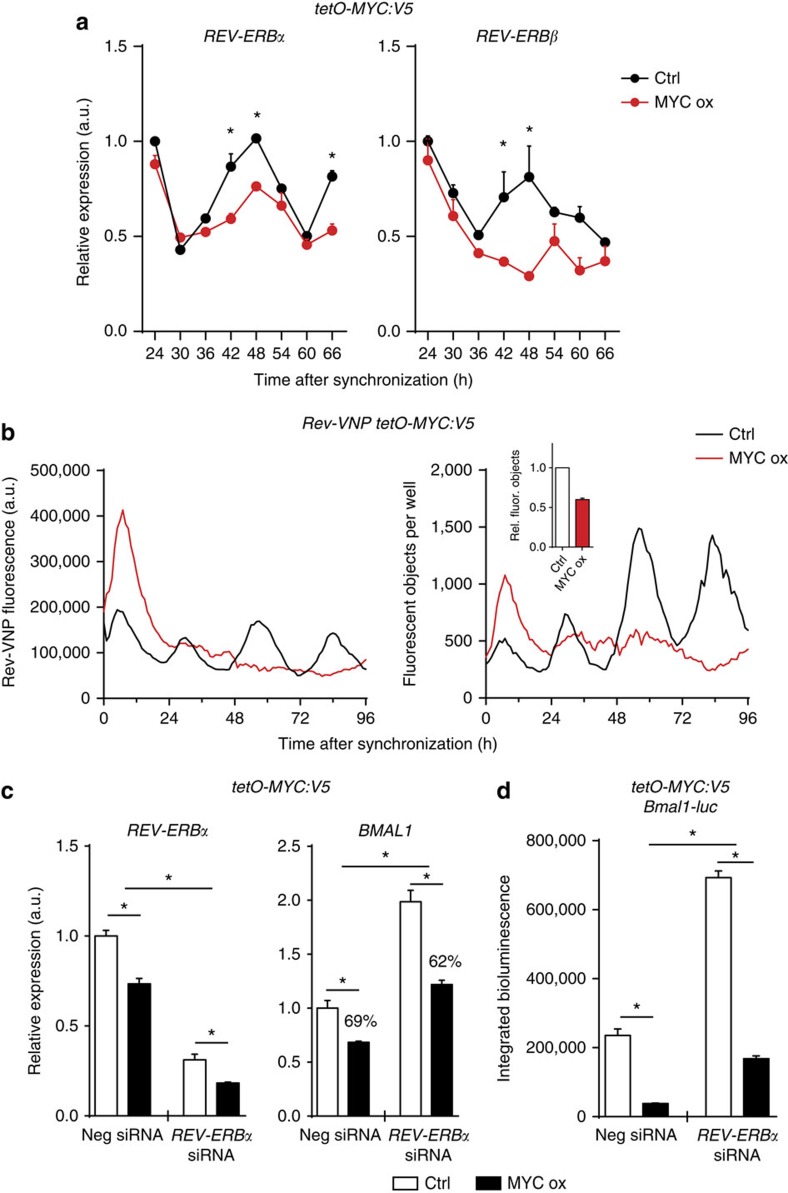
MYC represses *BMAL1* independent of REV-ERBα. (**a**) Quantitative PCR (qPCR) analysis of circadian expression profiles of *REV-ERBα* and *REV-ERBβ* transcripts in synchronized U2OS *t-rex tetO-MYC:V5* cells (*n*=3). (**b**) Total fluorescence and fluorescent objects quantified from synchronized U2OS *t-rex Rev-VNP tetO-MYC:V5* cells (*n*=1). Inset: normalized number of fluorescent objects (24–96 h integration; *n*=2). (**c**) qPCR analysis of *REV-ERBα* and *BMAL1* transcripts in siRNA-transfected U2OS *t-rex tetO-MYC:V5* cells (*n*=3). Unsynchronized cells were collected 24 h after doxycycline induction. (**d**) Integrated bioluminescence of *Bmal1-luc* transfected U2OS *t-rex tetO-MYC:V5* cells. Cells were transfected with indicated siRNAs 2 days before synchronization. Average expression levels (area under the curve) were determined over 72 h (*n*=3). Data are presented as mean±s.e.m. *P<0.05; two-way analysis of variance (ANOVA) with Bonferroni post-test.

We therefore asked whether MYC activates *REV-ERBα* in U2OS cells. Circadian expression profiles revealed that levels of *REV-ERBα*, as well as of *REV-ERBβ*, were downregulated rather than activated by overexpressed MYC (Fig. 2a). The data are in agreement with the dominant negative effect of MYC/MAX over CLOCK/BMAL1 in transient expression of *REVERBα-luc* (Supplementary Fig. 1b).

To analyse the impact of MYC on *REV-ERBα* in more detail we constructed a MYC-overexpressing cell line (U2OS *t-rex tetO-MYC:V5*) that expresses under control of the *Rev-erbα* promoter a destabilized nuclear venus reporter (*Rev-VNP*)^16^. *Rev-VNP* was rhythmically expressed as assessed by quantifying the total fluorescence and the number of fluorescent objects (cells) per well (Fig. 2b, black curves). Overexpression of MYC triggered a transient activation of *Rev-VNP* with a peak ∼7 h after induction, which was followed by downregulation and blunting of the circadian *Rev-VNP* rhythm (Fig. 2b, red curves).

We then integrated over a 3-day time course (24–96 h) the total number of fluorescent objects (cells expressing *Rev-VNP* above threshold) to assess whether the MYC-dependent loss of rhythmicity in the population of cells was due to desynchronization (same number of fluorescent objects in presence and absence of MYC) or due to an attenuated rhythm, that is, a reduction of the number of highly rhythmic cells (less fluorescent objects above threshold). One day after induction of MYC:V5, the average number of fluorescent objects was ∼2-fold reduced, indicating a decrease of the number of highly rhythmic cells rather than desynchronization of the population of cells (Fig. 2b, inset). MYC-dependent attenuation of the circadian *Rev-VNP* rhythm rather than MYC-induced desynchronization was also obvious on the level of single cells (Supplementary Movie 1). The data suggest that induction of MYC:V5 does not trigger a sustained overexpression of *REV-ERBα* but rather reduces its expression level and rhythm. Corresponding results were obtained with a *REVERBα-luc* reporter (Supplementary Fig. 2b).

Altman *et al*.^12^ reported induction of *REV-ERBα* in response to activation of MYC. However, they quantified *REV-ERBα* at a single time point early after induction of MYC (24 h). At this time *REV-ERBα* levels may still be slightly elevated due to its transient induction peaking after 7 h (Fig. 2b).

We thus knocked down *REV-ERBα* by short interfering RNA (siRNA) to assess whether the MYC-induced downregulation of *BMAL1* was dependent on REV-ERBα. *REV-ERBα* was ∼3-fold downregulated by specific versus control siRNA (Fig. 2c). When MYC was overexpressed, *REV-ERBα* levels were additionally reduced by 40% in presence of *REV-ERBα* specific siRNA and by 25% in presence of control siRNA (Fig. 2c). Downregulation of *REV-ERBα* by siRNA resulted in elevated expression of *BMAL1* (Fig. 2c), indicating that REV-ERBα is a potent repressor of *BMAL1* in U2OS cells. However, overexpressed MYC repressed *BMAL1* in both, *REV-ERBα* depleted and control cells (62 and 69%, respectively), indicating that MYC acted independent of *REV-ERBα*. Corresponding results were obtained with a *Bmal1-luc* reporter (Fig. 2d).

Downregulation of the positive limb of the circadian feedback loop has been shown to be sufficient to disrupt the molecular clock in U2OS cells^17^. Accordingly, the MYC-dependent attenuation of expression level and circadian rhythm of *Bmal1-luc* was partially rescued by constitutive overexpression of *BMAL1* together with *CLOCK* (Supplementary Fig. 2c,d). Together, these data suggest that overexpression of MYC did not enhance expression of *REV-ERBα* in a sustained manner. Rather, overexpressed MYC compromised the circadian system primarily via downregulation of *BMAL1*, *CLOCK* and its homologue *NPAS2* by a pathway independent of REV-ERBα and REV-ERBβ.

### MYC represses the circadian clock via interaction with MIZ1

**Figure 3:**
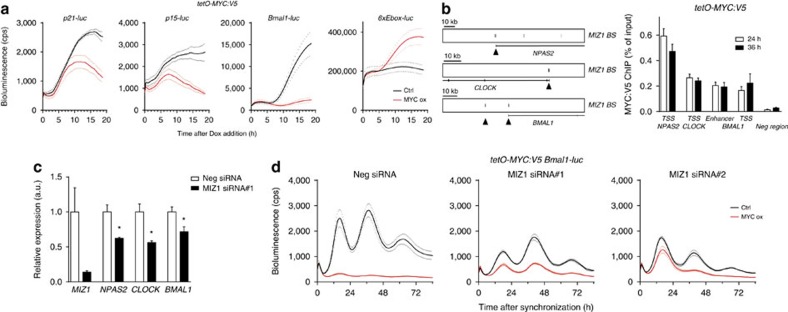
MYC directly represses clock genes via MIZ1. (**a**) Kinetics of MYC-dependent repression of *p21-luc*, *p15-luc* and *Bmal1-luc* and activation of *6xEbox-luc* (*n*=3). Unsynchronized U2OS *t-rex tetO-MYC:V5* cells were transiently transfected with the indicated reporter plasmids. At time point 0 MYC:V5 expression was either induced with doxycycline (MYC ox) or not induced with PBS (Ctrl). (**b**) Left panel: schematic of MIZ1 binding sites in *NPAS2*, *CLOCK* and *BMAL1* based on data from Walz *et al*.^14^. Black triangles indicate regions amplified in ChIP-PCR analysis. Right panel: ChIP-PCR analysis showing recruitment of induced MYC:V5 to MIZ1 binding sites in *NPAS2*, *CLOCK* and *BMAL1* at 24 and 36 h after synchronization of U2OS *t-rex tetO-MYC:V5* cells (*n*=3). (**c**) Downregulation of *MIZ1* with siRNA reduces expression of *NPAS2*, *CLOCK* and *BMAL1* in U2OS cells (*n*=3). Transcript levels of the indicated genes were determined by quantitative PCR (qPCR). (**d**) MYC-induced attenuation of the circadian clock is rescued by downregulation of MIZ1 (*n*=3). Synchronized U2OS *t-rex tetO-MYC:V5 Bmal1-luc* cells were transfected with *MIZ1* or control siRNAs. Note that downregulation of *MIZ1* attenuates the circadian rhythm of *Bmal1-luc* and lengthens the period (Supplementary Fig. 3a,b). Data are presented as mean±s.e.m. *P<0.05; Student's *t*-test.

MYC is not only a transcription activator. When expressed at high level, MYC has the potential to form a repressive complex with MIZ1 (ZBTB17) and downregulate in E-box-independent fashion expression of MIZ1 target genes such as the cyclin-dependent kinase inhibitor genes *p15* and *p21* (refs 14, 18, 19). We followed the MYC-dependent temporal expression profiles of luciferase reporter constructs of established and putative MIZ1 target genes. After induction of MYC:V5 with doxycycline, *Bmal1-luc* was repressed with similar kinetics as *p15-luc* and *p21-luc*, while *6xEbox-luc* was, as expected, induced when MYC was overexpressed (Fig. 3a). Examining a published ChIP-seq analysis of MIZ1 in U2OS cells^14^, we realized that MIZ1 binds to the promoters of *BMAL1, CLOCK* and *NPAS2* (Fig. 3b, left panel). We therefore performed a ChIP-PCR analysis and found that overexpressed MYC:V5 was recruited to the MIZ1 sites in *BMAL1*, *CLOCK* and *NPAS2* (Fig. 3b, right panel), suggesting that MYC binds to and represses these genes via MIZ1.

When MYC is not overexpressed MIZ1 acts as a transcription activator^20^. To assess whether MIZ1 supports expression of *BMAL1*, *CLOCK* and *NPAS2* in U2OS cells, we depleted *MIZ1* with siRNA (Fig. 3c). Expression of *BMAL1*, *CLOCK* and *NPAS2* was reduced indicating that MIZ1 contributes to the transcriptional activation of these clock genes. When MIZ1 was depleted by siRNAs, the circadian expression rhythm of *Bmal1-luc* was weakened in comparison to a treatment with control siRNA (Fig. 3d, black curves) and the circadian period was lengthened (Supplementary Fig. 3a). To assess whether the MYC-dependent disruption of the clock requires MIZ1, we induced MYC:V5 in MIZ1-depleted and control-treated cells (Fig. 3d, red curves). As expected, the circadian expression rhythm of *Bmal1-luc* was abolished when MYC:V5 was overexpressed in control-treated cells (Fig. 3d, red curve left panel). Concomitant downregulation of MIZ1, however, partially restored the expression level and circadian rhythms of *Bmal1-luc* despite overexpression of MYC:V5 (Fig. 3d, red curves middle and right panels). Correspondingly, the rhythm of *6xEbox-luc* was severely attenuated by induced MYC:V5 and fully rescued by siRNA depletion of *MIZ1* (Supplementary Fig 3b). Together the data indicate that overexpressed MYC represses *BMAL1* directly via MIZ1-dependent recruitment of MYC to MIZ1 binding sites.

### MYC-mediated attenuation of the clock requires MIZ1

**Figure 4:**
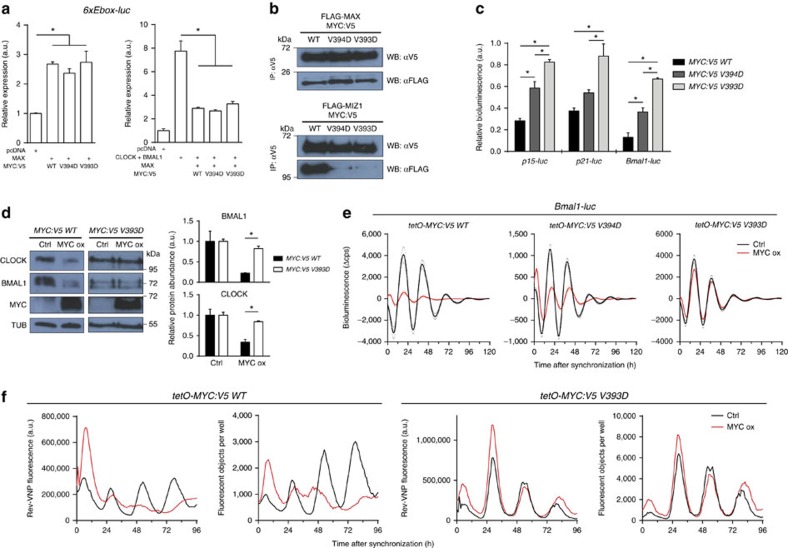
MYC mutants compromised in MIZ1 interaction do not disrupt the circadian clock. (**a**) Transactivation assay in HEK293 cells showing that MYC:V5 WT, V394D and V393D equally induce *6xEbox-luc* expression and compete with CLOCK/BMAL1 in a dominant negative manner (*n*=3). (**b**) Anti-V5 immunoprecipitation of MYC:V5 versions in HEK293 lysates showing that MYC:V5 V394D and V393D interact with MAX (upper panel) but not with MIZ1 (lower panel). Co-immunoprecipitation (Co-IP) of FLAG-tagged MAX and MIZ1 was detected with anti-FLAG antibodies. Refer to Supplementary Fig. 4a for inputs and reciprocal anti-FLAG co-IP's. (**c**) Overexpressed MYC:V5 V394D and V393D are inefficient in repression of MIZ1 target genes. Expression of transiently transfected *p15-luc*, *p21-luc* and *Bmal1-luc* reporters in U2OS *t-rex tetO-MYC:V5* cells expressing the indicated versions of MYC. Bioluminescence was quantified 18 h after MYC induction with doxycycline and normalized to PBS-treated samples (*n*=3). (**d**) Western blot analysis (left) and densitometric protein quantification (right) of CLOCK and BMAL1 in U2OS cells overexpressing MYC and MYC V393D 24 h after doxycycline induction (*n*=3). (**e**) Baseline-subtracted bioluminescent traces from U2OS *t-rex tetO-MYC:V5 WT*, *V394D* and *V393D* cells transiently transfected with *Bmal1-luc* (*n*=3). For the raw data refer to Supplementary Fig. 4d. (**f**) Total fluorescence and fluorescent objects quantified from synchronized U2OS *t-rex Rev-VNP* cells stably transfected with inducible *MYC:V5 WT* and *V393D* (*n*=1). Data are presented as mean±s.e.m. *P<0.05; one-way analysis of variance (ANOVA) with Bonferroni post-test.

To challenge this hypothesis, we generated the V5-tagged MYC V394D mutant, which is impaired in its interaction with MIZ1 (ref. 21) and in addition the variant V393D. ChIP-PCR analysis demonstrated that, as was shown for V394D^21^, the newly generated V393D variant binds with similar affinity to E-boxes as WT MYC (Supplementary Fig. 4a). Both mutant versions of MYC:V5 induced expression of *6xEbox-luc* in HEK293 cells and interfered in dominant negative fashion with CLOCK/BMAL1-dependent activation of *6xEbox-luc* to the same extent as wild-type MYC:V5 (Fig. 4a). Theses data suggest that the E-box-dependent functionality of MYC:V5 V394D and MYC:V5 V393D was not affected. Both mutant versions interacted with MAX with similar efficiency as MYC:V5 (Fig. 4b, upper panel; and Supplementary Fig. 4b). In contrast, the V394D and V393D variants showed markedly reduced association with MIZ1 (Fig. 4b, lower panel; and Supplementary Fig. 4b). Next, we produced stable U2OS cell lines harbouring doxycycline-inducible *MYC:V5 V394D* and *MYC:V5 V393D* genes. Both inducible transcripts accumulated to similar levels as *MYC:V5* (Supplementary Fig. 4c). However, repression of *p15-luc, p21-luc* as well as *Bmal1-luc* reporters was alleviated in the cell lines expressing the V393D and V394D versions of MYC:V5 (Fig. 4c). The levels of CLOCK and BMAL1 proteins were strongly reduced by overexpression of MYC:V5, but remained essentially unaffected by induction of MYC:V5 V393D (Fig. 4d). Circadian expression rhythms of *Bmal1-luc* and *6xEbox-luc* reporters were strongly attenuated by overexpression of wild-type MYC:V5, but only mildly affected by overexpression of V394D and essentially unaffected by the V393D version (Fig. 4e and Supplementary Fig. 4d). Similarly, *Rev-VNP* (ref. 16) expression levels and rhythms were strongly attenuated by overexpressed MYC:V5 but unaffected by induction of MYC:V5 V393D (Fig. 4f and Supplementary Movies 1 and 2). The data demonstrate that overexpressed MYC attenuates the circadian clock via MIZ1-mediated E-box-independent repression.

### MYC-dependent cell cycle stimulation requires MIZ1

**Figure 5:**
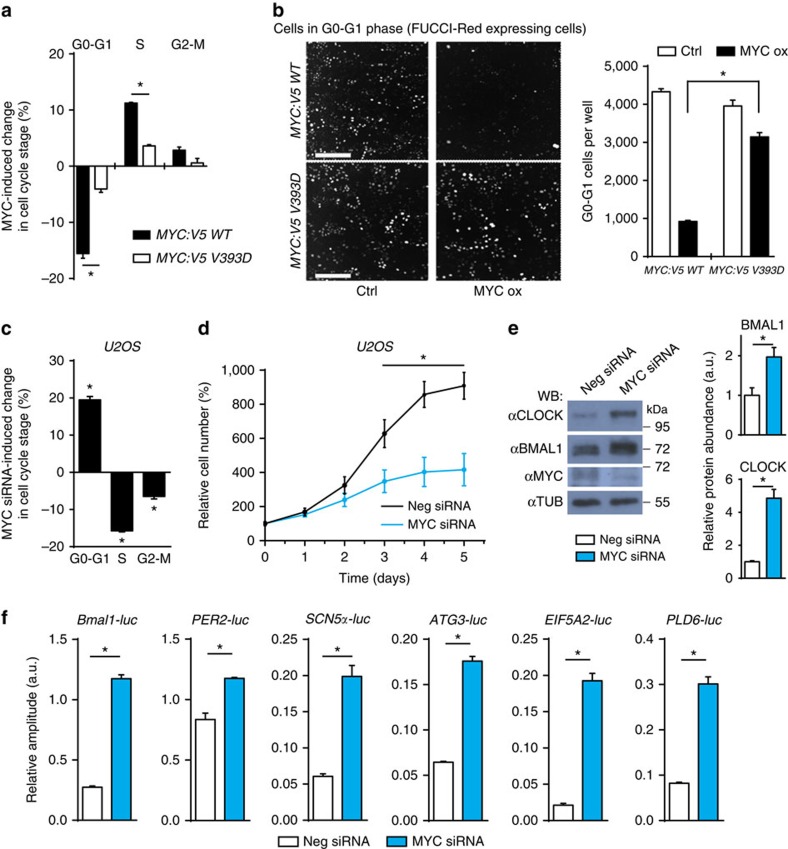
MYC inversely regulates the circadian clock and proliferation. (**a**) *MYC:V5 WT* and *V393D* U2OS cells were stained with propidium iodide 48 h after induction and DNA content was quantified by FACS (*n*=3). Values indicate difference (in %) to PBS-treated cells in the respective cell cycle phase. (**b**) Left panel: fluorescence microscopy of U2OS *t-rex tetO*-*MYC:V5* cells stably expressing mCherry-Cdt1 (FUCCI-Red G1 marker) 48 h after treatment with doxycycline to induce MYC:V5 (MYC ox) or PBS (Ctrl). Scale bar, 300 μm. Right panel: quantification of cells in G0 or G1 phase by mCherry-Cdt1 expression (*n*=3) (**c**) FACS analysis of U2OS cells stained with propidium iodide 48 h after transfection with MYC siRNA (*n*=3). Values indicate difference to cells transfected with negative siRNA. (**d**) Growth curve of U2OS cells transfected with MYC siRNA and negative siRNA (*n*=3). (**e**) Western blot analysis (left) and densitometric protein quantification (right) showing that MYC depletion by siRNA supports increased BMAL1 and CLOCK expression in U2OS cells (*n*=3). (**f**) Relative amplitudes (ChronoStar software) of circadian luciferase rhythms of indicated U2OS reporter cell lines (*n*=3). The cells were transfected with MYC siRNA and negative siRNA as indicated. Data are presented as mean±s.e.m. *P<0.05; Student's *t*-test (**e,f**), one-way (**b**,**c**) and two-way (**a**,**d**) analysis of variance (ANOVA) with Bonferroni post-test.

Elevated MYC expression is considered to drive cell growth and proliferation through genome-wide interference with physiological regulation of E-box-dependent transcription^22^. Since the E-box-dependent activation properties are preserved in the V393D version of MYC:V5 (Fig. 4a), we asked how it affects cell growth and proliferation. Consistent with previous studies^21^, induction of MYC:V5 reduced the fraction of cells in G1 phase and increased the proportion of cells in S, G2 and M phases (Fig. 5a,b). In contrast, overexpression of MYC:V5 V393D did not support proliferation to a substantial extent. These data suggest that stimulation of proliferation of U2OS cells by MYC is crucially dependent on gene repression via MIZ1. Together the data suggest that overexpression of MYC supports proliferation and attenuates the circadian clock predominantly via MIZ1.

### Knockdown of MYC induces circadian amplitude

Next, we analysed effects of MYC depletion. Knockdown of MYC by siRNA resulted in decreased proliferation of U2OS cells (Fig. 5c,d). Concomitantly, expression levels of BMAL1 and CLOCK increased (Fig. 5e). Furthermore, the relative amplitudes (the oscillation amplitude divided by the mean expression) of the circadian expression rhythms of *Bmal1-luc* and other circadian reporters significantly improved in cells transfected with MYC siRNA (Fig. 5f and Supplementary Fig. 5a). Corresponding effects were observed in U2OS cells expressing *Rev-VNP* (Supplementary Fig. 5b, Supplementary Movie 3). Thus, downregulation of MYC with siRNA increased the relative amplitude of the *Rev-VNP* rhythm. In summary, the data indicate that downregulation of MYC attenuates proliferation and strengthens the circadian clock.

### Inverse correlation of MYC and clock genes in lymphoma

**Figure 6:**
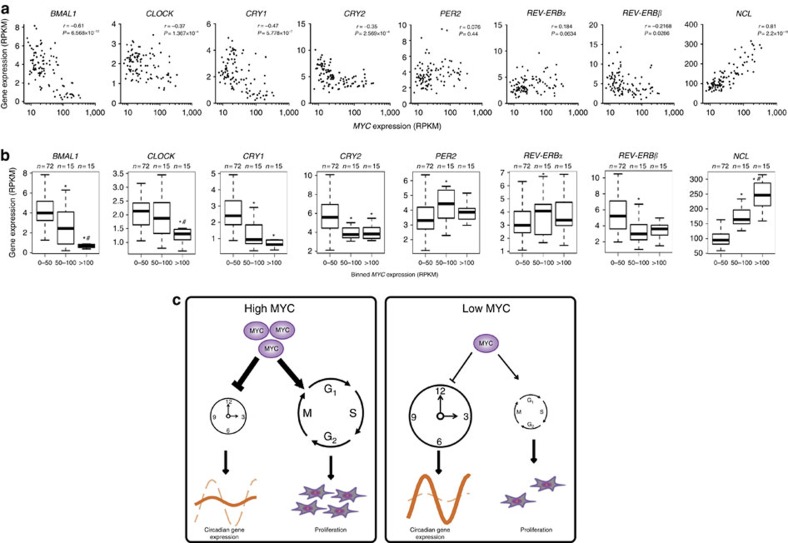
*MYC* inversely correlates with *BMAL1* expression in human lymphomas. (**a**) Scatter plots of expression levels of *MYC* versus the indicated clock genes in 102 human lymphoma samples of the ICGC MMML-Seq project^37^ (RPKM: log2 sequence reads per kilobase transcript per million reads^38^). Expression of *MYC* versus its established target *NUCLEOLIN* (*NCL*) is shown as a positive control. (**b**) Data were binned according to the indicated MYC expression levels. Clock gene expression levels are shown by Box-plots. Significant differences (*P*<0.05; Student's *t*-test) of expression levels relative to box 1 (0–50) and box 2 (50–100) are indicated by asterisks (*) and number signs (#), respectively. (**c**) Model of the coordinating function of MYC. Left panel: high levels of MYC suppress the circadian clock by MIZ1-dependent downregulation of BMAL1/CLOCK (see text), which results in low amplitude expression rhythms of clock-controlled genes. On the other hand, high MYC levels support cell growth and proliferation (for example, by inhibition of *p15* and *p21*, see text). Right panel: at low levels of MYC, the circadian clock is not inhibited and supports high amplitude expression rhythms of clock-controlled genes. Low levels of MYC do not support cell growth and proliferation.

MYC is overexpressed in many tumours and depletion of MYC is known to detain tumour growth^22^. Encouraged by the inverse correlation of MYC with BMAL1 and CLOCK expression in U2OS cells, we assessed expression of these genes in human lymphoma by analysing RNA-seq data of 102 patient samples. Lymphoma was chosen since this type of malignancy requires interaction of MYC and MIZ1 (ref. 23). Consistent with our observation in U2OS cells, expression of both clock genes correlated inversely with *MYC* levels in human lymphoma (Pearson coefficient −0.61 and −0.37 for *BMAL1* and *CLOCK*, respectively; *n*=102), whereas MYC-activated genes such as *NCL* and *NCLN* showed a positive correlation (Fig. 6a,b and Supplementary Fig. 6). MYC levels did not correlate positively with expression of circadian genes regulated via E-boxes. Particularly, *REV-ERBα* and *β* did not significantly correlate with *MYC*.

The circadian clock and cell cycle/proliferation are major programs controlling expression of specific, potentially overlapping sets of genes^24^,^25^,^26^,^27^. Since the simultaneous regulatory activity of both programs in the same cell may create conflicting signals, it seems conceivable that their activity is coordinated under physiological conditions. It has been shown that in regenerating mouse liver, the circadian clock gates the cell cycle^28^ and that in NIH3T3 cells the cell cycle resets the circadian clock^29^,^30^. The circadian clock in the liver of a living animal oscillates with high amplitude, while the circadian clock in isolated cells appears to be substantially weaker^13^,^31^. Hence, the dominance of one cycling programme over the other may depend on their relative strength or rhythmic momentum. Our observations suggest that MYC is a master regulator coordinating both programs. Expression levels of MYC determine the relative strength of the circadian clock versus cell cycle/proliferation and hence, their impact on gene expression and cellular physiology. MYC attenuates the circadian system and promotes proliferation. Both functions are critically dependent on complexes of MYC with MIZ1. Such repressive complexes form preferentially at high MYC levels^32^. The inverse correlation of *BMAL1* versus *MYC* expression levels in human lymphomas and the absence of a positive correlation of MYC with circadian E-box genes support this hypothesis. The correlation suggests that circadian physiology might be compromised in tumours with amplified *MYC*. Strategies targeting the interaction of MYC with MIZ1 could help to recover circadian control over cellular physiology in malignant cells and potentially inhibit uncontrolled growth without major effects on differentiated post-mitotic cells with low *MYC* expression.

It seems conceivable that rhythmic downregulation of metabolic and biosynthetic functions by the circadian clock may be in conflict with rapid cell growth and proliferation. Hence, aside from malignant conditions, MYC may also coordinate the relative impact on gene expression of the circadian clock versus cell cycle/proliferation under physiological conditions such as in the developing and differentiating embryo.

## Methods

### Cell culture and transfections

U2OS *t-rex* (T-Rex, Life Technologies) and HEK293T (ATCC) cells were maintained in DMEM supplemented with 10% fetal bovine serum (FBS) and 1xPenStrep. Cell culture reagents were obtained from Life Technologies unless indicated differently. U2OStx were transfected with *AhdI*-linearized pcDNA4/TO vectors containing different alleles of human MYC using Xfect (Clontech) and stable transfectants were selected using growth medium supplemented with 50 μg ml^−1^ hygromycin and 100 μg ml^−1^ zeocin (InvivoGen). Positive clones were subsequently, transiently or stably transfected with circadian reporter constructs using Xfect reagent and, if applicable, selected with 1 μg ml^−1^ puromycin (InvivoGen). U2OS stable cell lines expressing the promoter-luciferase constructs were described previously^13^. For RNAi experiments, U2OS cells were transfected with the indicated siRNA (sequences are given in Supplementary Table 1) using lipofectamine RNAiMAX reagent according to manufacturer's protocols. For luciferase reporter assays, HEK293T cells were transfected with the indicated constructs using Lipofectamine2000 and 24 h later luciferase expression was assayed using Dual-luciferase Reporter Assay (Promega) and an EnSpire Reader (Perkin Elmer).

### Plasmid constructs

*Bmal1-luc* and *6xEbox-luc* vectors were kindly provided by Dr Steven A Brown and Dr Achim Kramer^33^,^34^. *p15-luc*, *p21-luc* and *MIZ1* open reading frame (ORF) vectors were kindly provided by Dr Elmar Wolf. Rev-VNP fluorescent reporter containing 13 kb of murine *Rev-erbα* promoter coupled to *Venus-NLS-PEST* was kindly provided by Dr Ueli Schibler. Promoter sequences (1 kb) of *PER2, REV-ERBα, SCN5α* and *GAPDH* genes cloned in pGL4.20puro vector were described previously^13^. *BMAL1*, *CLOCK* and *MAX* ORFs were amplified from U2OS complementary DNA (cDNA) and cloned in pcDNA4/TO vector. The *MYC* and *FLAG:REV-ERBα* ORFs were obtained from Addgene and subcloned in pcDNA4/TO. *MYC* V394D and V393D mutations were performed using DF-Pfu polymerase (Bioron). Cloning and mutagenesis primers are available on request.

### Real-time bioluminescence monitoring

For RNAi or transfection experiments, cells were seeded into a 96-well plate (20,000–30,000 cells per well) and next day transfected with the indicated siRNAs. 24 h later the transfection medium was removed and cells were synchronized with 1 μM dexamethasone for 20 min and washed with PBS. After addition of warm luminescene medium (DMEM w/o Phenolred (PAA) supplemented with 10% FBS, 25 mM Hepes, 1xPenStrep, and 0.125 μM luciferin (BioSynth), 10ng ml^−1^ doxycycline) the plate was sealed and bioluminescence was recorded for 30 min intervals at 37 °C with an EnSpire Reader (Perkin Elmer). Circadian period and amplitude analysis was performed using ChronoStar software^35^.

### Gene expression analysis

Transfected or synchronized cells were lysed with TriFaster (GeneON) and total RNA was extracted according to manufacturer's protocol. cDNA was synthesized with Maxima First Strand cDNA Synthesis Kit (Thermo Scientific). Quantitative PCR was performed using GoTaq Master Mix (Promega) and LightCycler 480 (Roche) and relative gene expression was quantified using a ΔΔCt method with *GAPDH* as a reference gene. Primer sequences are listed in Supplementary Table 1.

### Chromatin immunoprecipitation (ChIP)

U2OS *t-rex* cells were collected 24 and 36 h after synchronization (10 ng ml^−1^ doxycycline added at time point 0) and immediately cross-linked in 1% formaldehyde for 10 min. ChIP was performed as described previously with minor modifications^36^. In brief, pelleted nuclei were suspended in 300 μl IP buffer (150 mM NaCl, 5 mM EDTA, NP-40 (0.5%), Triton X100 (1.0%), 50 mM Tris-HCl, pH 7.5) supplemented with 0.1% SDS and sonicated (30 s on/30 s off cycles for 20 min) using a Bioruptor (Diagenode Inc.). Sheared chromatin (equivalent of 10^6^ cells) was incubated overnight at 4 °C with 3 μl of anti-BMAL1 (ref. 13; 0.1 mg ml^−1^) and 0.5 μl anti-V5 antibodies (1 mg ml^−1^, 46-0705, Life Technologies), and obtained immune complexes were precipitated with salmon sperm DNA blocked protein A-agarose beads (Millipore) equilibrated five times with IP buffer. Precipitated DNA was recovered by boiling for 10 min in 10% Chelex slurry (Bio-Rad) followed by Proteinase K (150 μg ml^−1^, New England Biolabs) treatment at 55 °C for 30 min. Proteinase K was subsequently inhibited by boiling at 95 °C for 10 min and beads were removed by centrifugation at 12,000*g* at 4 °C. DNA-containing supernatants were analysed by quantitative PCR, and values were normalized to percentage of input. Primer sequences are listed in Supplementary Table 1.

### Cell proliferation assays

Prior to siRNA transfection, U2OS *t-rex* cells were seeded on 96-well plates (5,000 cells per well) in 100 μl DMEM (10% FBS, 1xPenStrep). Twenty four hours after transfection (day 0), medium was replaced with 100 μl DMEM (10% FBS, 1xPenStrep). Cell number was measured daily by incubating cells for 1.5 h with DMEM containing 1/10 diluted WST-8 reagent at 37 °C (Cell Counting Kit-8, Sigma-Aldrich) and absorbance was read at 450 nm using an EnSpire Reader (Perkin Elmer).

### Fluorescent microscopy

U2OS *t-rex Rev-VNP* (*tetO-MYC WT/V393D* or siRNA transfected) cells were seeded (10,000 cells per well) on ImageLock 96-well plates (Essen Bioscience) in 100 μl DMEM (10% FBS, 1xPenStrep). Next day, after dexamethasone synchronization and PBS wash, cells were incubated in 100 μl PBS- or Doxycycline-containing DMEM (10% FBS, 1xPenStrep) and monitored with IncuCyte ZOOM reader (Essen Bioscience). To detect cells in G0–G1 phase, U2OS overexpressing *MYC* alleles were stably transfected with FUCCI-Red construct and puromycin-resistant clones were selected. Then 7,000 cells were seeded on ImageLock 96-well plates (Essen Bioscience) and next day, after addition of doxycycline, cells were monitored with an IncuCyte ZOOM reader (Essen Bioscience) and red-fluorescent objects were counted using in-built software. Total fluorescence and number of fluorescent objects were measured using in-built software.

### FACS

After 3 days of doxycycline induction, trypsinized and PBS-washed cells (∼10^6^ cells) were fixed in 70% ethanol for 1 h at 4 °C. After centrifugation for 5 min at 300*g*, cells were washed once with PBS and incubated for 10 min at room temperature in 1 ml propidium iodide-staining solution (PBS, 0.1% TritonX-100, 10 μg ml^−1^ freshly added propidium iodide, 100 μg ml^−1^ freshly added RNaseA). Cellular DNA content was measured with a FACSCanto analyzer (BD).

### Co-immunoprecipitation (Co-IP) and western blotting

For protein analysis, synchronized U2OS cells were lysed in ice-cold lysis buffer (25 mM Tris-HCl, pH 8.0, 150 mM NaCl, 0.5% Triton X100, 2 mM EDTA, 1 mM NaF and protease inhibitor cocktail (Roche, 04693159001)) for 10 min on ice and sonicated in ice water in an ultrasonic bath (Merck) for 10 min. Then lysates were pre-cleared from cell debris by centrifugation at 16,000*g* for 15 min at 4 °C and the protein concentration in the supernatant was determined with Roti-Quant (Carl Roth). Samples (200 μg of total protein) were boiled with the appropriate amount of 4x Laemmli buffer (250 mM Tris-HCl pH 6.8, 6% SDS, 40% Glycerol, 0.04% Bromphenolblue and 20% Mercaptoethanol) for 3 min at 95 °C and separated using 12% SDS-PAGE. After semi-dry transfer (PEQLAB) on nitrocellulose membranes (GE Healthcare), protein extracts were decorated with anti-BMAL1 (ref. 13; 1:750), anti-CLOCK (ref. 13; 1:500), anti-MYC (1:400, N-262, SantaCruz), anti-V5 (1:5,000, 46-0705, Life Technologies) and anti-FLAG (1:5,000, M2, Sigma-Aldrich) antibodies in 5% milk TBS at 4 °C overnight. Next day, membrane was washed three times in TBS and incubated with the appropriate HRP-conjugated secondary antibody in 5% milk TBS for 1 h at room temperature. After 4 TBS washes, the membrane was exposed to X-ray films (Super RX, FUJIFILM) and developed using an AGFA automatic processor developer. For co-IP, lysates from transfected HEK293T cells were prepared as described above. Lysates (500 μg total protein) were incubated with anti-V5 (0.5 μl) antibodies or 40 μl of PBS-washed anti-FLAG M2 Affinity Gel (Sigma-Aldrich) agitating at 4 °C overnight. Next day, 40 μl of PBS-washed protein A sepharose beads (GE Healthcare) were added to bind anti-V5 immune complexes and incubated for 2 h. Then beads were washed three times with PBS and protein was eluted by boiling in 4x Laemmli buffer. Precipitated proteins were analysed by western blotting as described above. Uncropped blots are shown in Supplementary Fig. 7.

## Additional information

**How to cite this article:** Shostak, A. *et al*. MYC/MIZ1-dependent gene repression inversely coordinates the circadian clock with cell cycle and proliferation. *Nat. Commun.* 7:11807 doi: 10.1038/ncomms11807 (2016).

## Supplementary Material

Supplementary Information Supplementary Figures 1-7 and Supplementary Table 1 and Supplementary References.

Supplementary Movie 1 Microscopic imaging of PBS (Ctrl) or doxycycline (MYC ox) treated U2OS t-rex Rev-VNP tetO-MYC:V5 WT cells after synchronization. Images were recorded hourly over a period of 4 days.

Supplementary Movie 2 Microscopic imaging of PBS (Ctrl) or doxycycline (MYC ox) treated U2OS t-rex Rev-VNP tetO-MYC:V5 V393D cells after synchronization. Images were recorded hourly over a period of 4 days.

Supplementary Movie 3 Microscopic imaging of neg. siRNA and MYC siRNA transfected U2OS t-rex Rev-VNP cells after synchronization. Images were recorded hourly over a period of more than 5 days.
