# Supplementary material for: MYC/MIZ1-dependent gene repression inversely coordinates the circadian clock with cell cycle and proliferation
Source: Nat Commun. 2016 Jun 24;7:11807. doi: 10.1038/ncomms11807 (PMC4931031; doi:10.1038/ncomms11807)
Supplement: Supplementary Information — Supplementary Figures 1-7 and Supplementary Table 1 and Supplementary References. [file ncomms11807-s1.pdf]

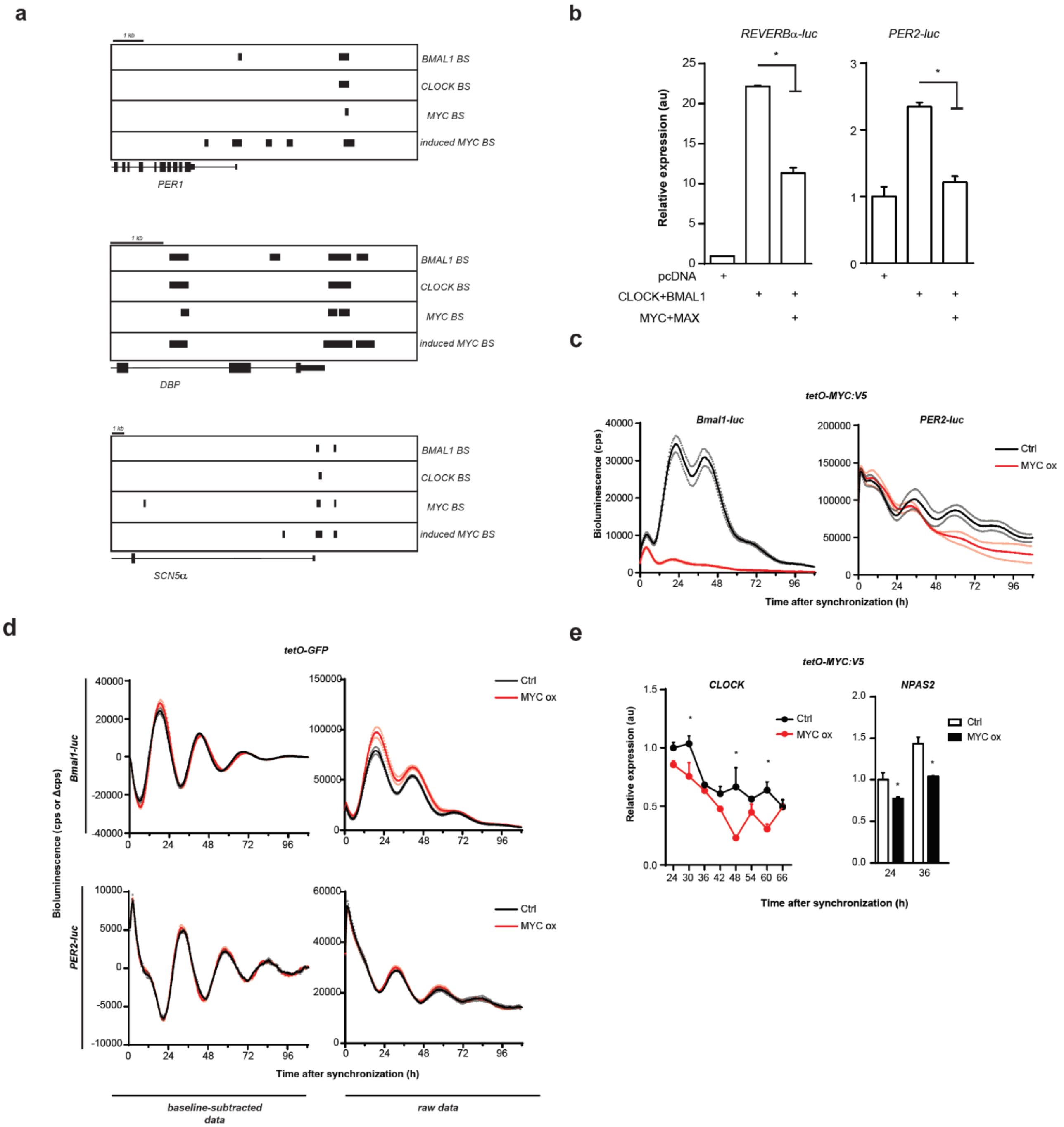
a
1 kb
BMAL1 BS
CLOCK BS
MYC BS
induced MYC BS
PER1
DBP
SCN5α
b
REVERBα-luc
PER2-luc
Relative expression (au)
pcDNA
CLOCK+BMAL1
MYC+MAX
c
tetO-MYC:V5
Bmal1-luc
PER2-luc
Bioluminescence (cps)
Ctrl
MYC ox
Time after synchronization (h)
d
tetO-GFP
Bmal1-luc
PER2-luc
Bioluminescence (cps or Δcps)
Time after synchronization (h)
baseline-subtracted data
raw data
e
tetO-MYC:V5
CLOCK
NPAS2
Relative expression (au)
Ctrl
MYC ox
Time after synchronization (h)

Supplementary Figure 1. Overexpression of MYC attenuates the circadian clock

(a) Genomic loci of indicated clock genes and clock-controlled genes with binding sites (BS) of BMAL1, CLOCK, endogenous MYC and, overexpressed MYC in U2OS cells (based on the data from[1,2]. **(b)** MYC/MAX restricts stronger induction of *REVERBα-luc* and *PER2-luc* by CLOCK/BMAL1 (n=3). HEK293 cells were transfected with equal amounts (30 ng) of each plasmid (*BMAL1*, *CLOCK*, *MYC* and *MAX*) and the indicated circadian *luc* reporter. **(c)** Raw bioluminescence signals from synchronized *Bmal1-luc* and *PER2-luc* U2OS *t-rex tetO-MYC:V5* cells (n=3). Baseline-subtracted traces are shown in Fig. 1g. **(d)** Control (expressing GFP instead of MYC) showing baseline-subtracted and raw bioluminescence from synchronized *Bmal1-luc* and *PER2-luc* U2OS *t-rex tetO-GFP* cells (n=3). **(e)** qPCR analysis of endogenous *CLOCK* and *NPAS2* transcripts in synchronized U2OS *t-rex tetO-MYC:V5* cells treated with doxycycline to induce MYC:V5 (MYC ox) or PBS (Ctrl) (n=3). Data are presented as mean ± SEM. * $P < 0.05$; one-way **(b)** and two-way **(e)** ANOVA with Bonferroni post-test.

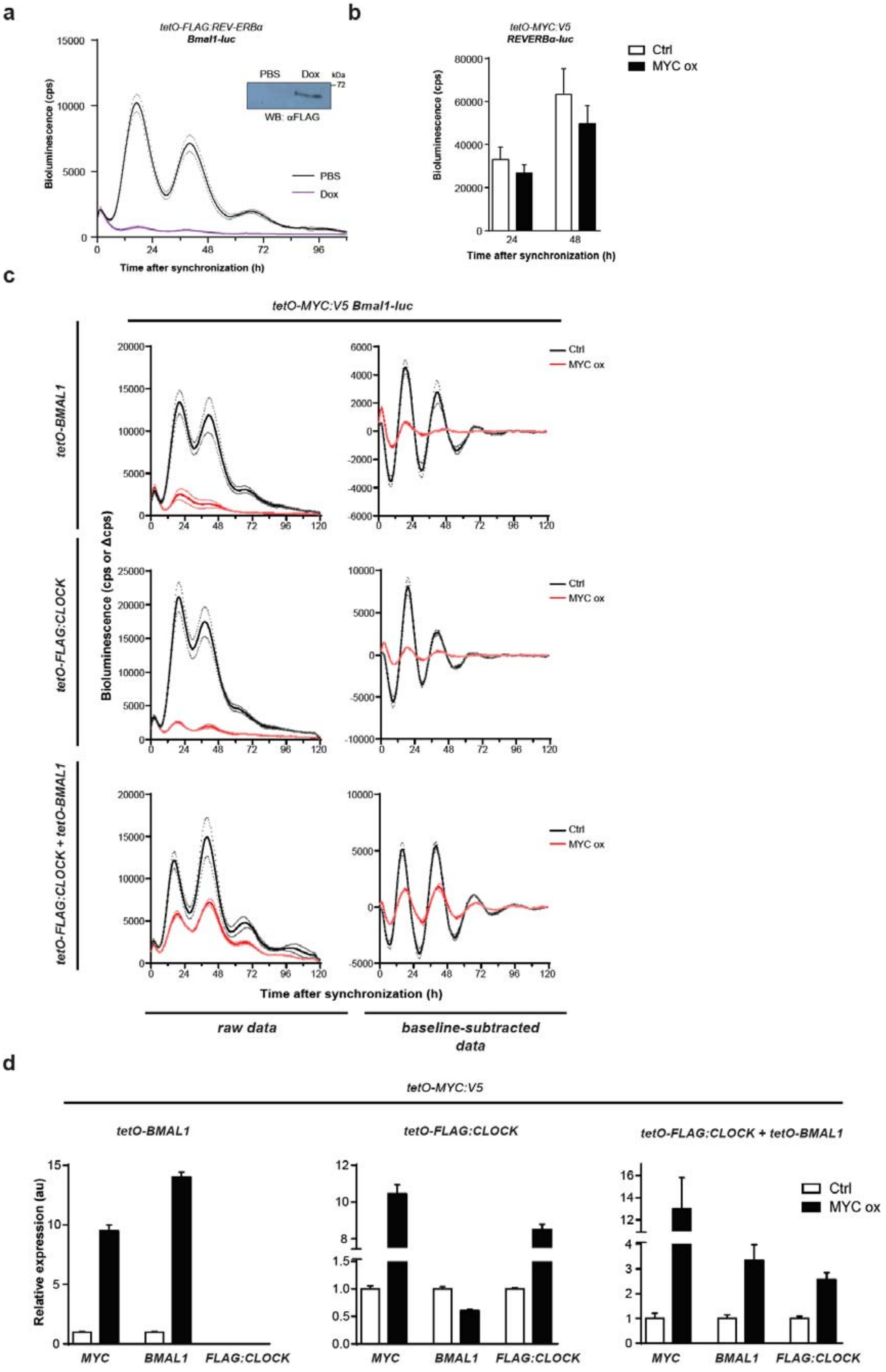
a
tetO-FLAG:REV-ERBα
Bmal1-luc
Bioluminescence (cps)
PBS
Dox
kDa
72
WB: αFLAG
Time after synchronization (h)
b
tetO-MYC:V5
REVERBα-luc
Ctrl
MYC ox
Bioluminescence (cps)
Time after synchronization (h)
c
tetO-MYC:V5 Bmal1-luc
tetO-BMAL1
tetO-FLAG:CLOCK
tetO-FLAG:CLOCK + tetO-BMAL1
Bioluminescence (cps or Δcps)
Ctrl
MYC ox
Time after synchronization (h)
raw data
baseline-subtracted data
d
tetO-MYC:V5
tetO-BMAL1
tetO-FLAG:CLOCK
tetO-FLAG:CLOCK + tetO-BMAL1
Relative expression (au)
Ctrl
MYC ox
MYC
BMAL1
FLAG:CLOCK

Supplementary Figure 2. MYC represses *BMAL1* independent of REV-ERBα

(a) Raw bioluminescence signals from synchronized *Bmal1-luc* transfected U2OS *t-rex tetO-FLAG:REV-ERBα* cells (n=3). FLAG:REV-ERBα expression was induced with doxycycline (Dox) or control-treated with PBS. Inset: Western Blot analysis showing expression of FLAG:REV-ERBα 24 hours after doxycycline induction. **(b)** Bioluminescence at 24 and 48 hours after synchronization of U2OS *t-rex tetO-MYC:V5* cells stably transfected with *REVERBα-luc* (n=12). **(c)** Constitutive overexpression of *BMAL1* together with *CLOCK* rescues MYC-induced attenuation of the circadian clock. Baseline-subtracted and raw bioluminescence from synchronized *Bmal1-luc* U2OS *t-rex tetO-MYC:V5* cells stably transfected with doxycycline-inducible *BMAL1, FLAG:CLOCK* and both genes (n=3). **(d)** qPCR analysis of *MYC*, *BMAL1* and *FLAG:CLOCK* transcripts 24 hours after induction of MYC together with *BMAL1, FLAG:CLOCK* and *FLAG:CLOCK* + *BMAL1* (n=3). Data are presented as mean ± SEM.

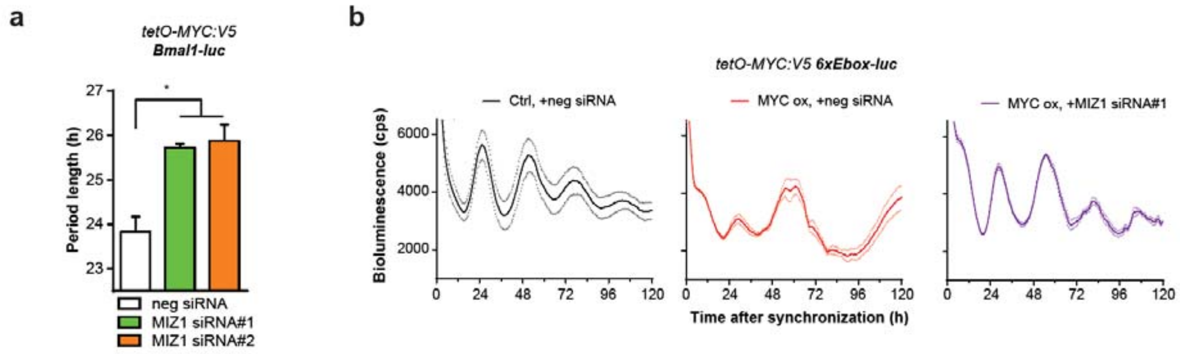

Supplementary Figure 3. MIZ1 knockdown attenuates MYC-mediated repression of the circadian clock

(a) Downregulation of MIZ1 causes period lengthening. Period analysis of PBS-treated (uninduced) U2OS *t-rex tetO-MYC:V5 Bmal1-luc* cells transfected with negative and MIZ1 siRNAs (n=3). **(b)** Downregulation of MIZ1 rescues MYC-induces attenuation of *6xEbox-luc* rhythm. Raw bioluminescence signals from synchronized U2OS *t-rex tetO-MYC:V5 6xEbox-luc* cells transfected with negative and MIZ1 siRNAs (n=3). Data are presented as mean ± SEM. * $P < 0.05$; Student's *t*-test.

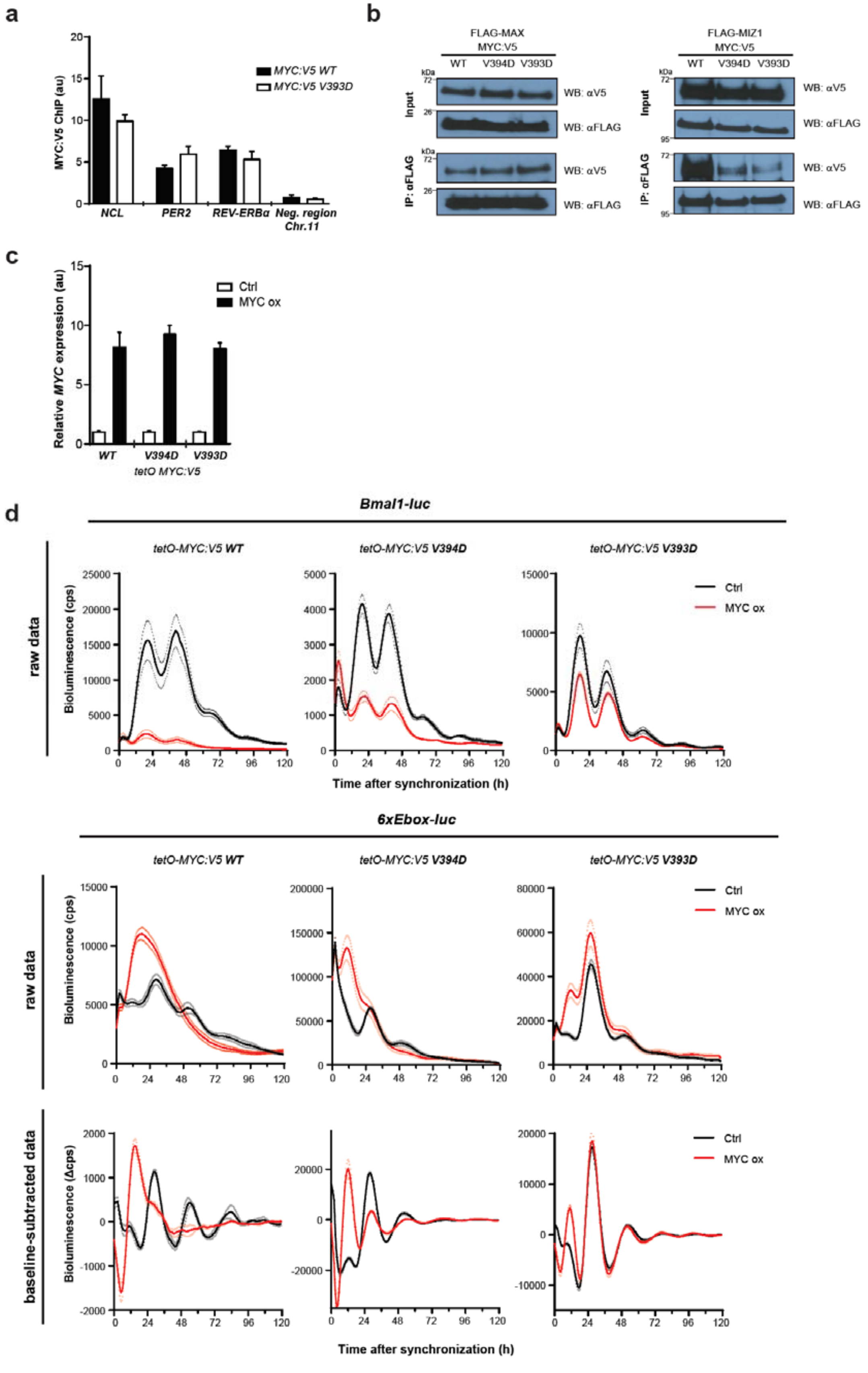
a
MYC:V5 WT
MYC:V5 V393D
MYC:V5 ChIP (au)
NCL
PER2
REV-ERBα
Neg. region Chr.11
b
FLAG-MAX
MYC:V5
FLAG-MIZ1
MYC:V5
WT
V394D
V393D
Input
IP: αFLAG
WB: αV5
WB: αFLAG
kDa
c
Ctrl
MYC ox
Relative MYC expression (au)
WT
V394D
V393D
tetO MYC:V5
d
Bmal1-luc
tetO-MYC:V5 WT
tetO-MYC:V5 V394D
tetO-MYC:V5 V393D
raw data
Bioluminescence (cps)
Time after synchronization (h)
6xEbox-luc
baseline-subtracted data
Bioluminescence (Δcps)

Supplementary Figure 4. MYC mutants compromised in MIZ1 binding do not disrupt the circadian clock

(a) ChIP-PCR analysis of MYC:V5 WT and V393D recruited to E-boxes in *NCL, PER2,* and *REV-ERBα* promoters in synchronized and doxycycline-induced U2OS *t-rex tetO-MYC:V5* cells (n=3). To obtain relative enrichment the doxycycline-ChIP signal was divided by PBS-ChIP signal for both cell genotypes. **(b)** Co-immunoprecipitation of the indicated MYC:V5 versions with FLAG-MAX or FLAG-MIZ1 in lysates of transfected HEK293 cells. Input and FLAG-IP was analyzed by Western blot with V5 and FLAG antibodies. **(c)** qPCR analysis of relative expression levels of WT and mutant *MYC:V5* genes in stably transfected U2OS *t-rex* cells (n=3). Samples were analyzed 24 hour after induction with doxycycline (MYC ox) or PBS treatment (Ctrl). **(d)** Bioluminescence recordings from synchronized *Bmal1-luc* and *6xEbox-luc* U2OS *t-rex tetO-MYC:V5* (n=3). Note: the baseline-subtracted *Bmal1-luc* traces are shown in Fig. 4e. Data are presented as mean ± SEM.

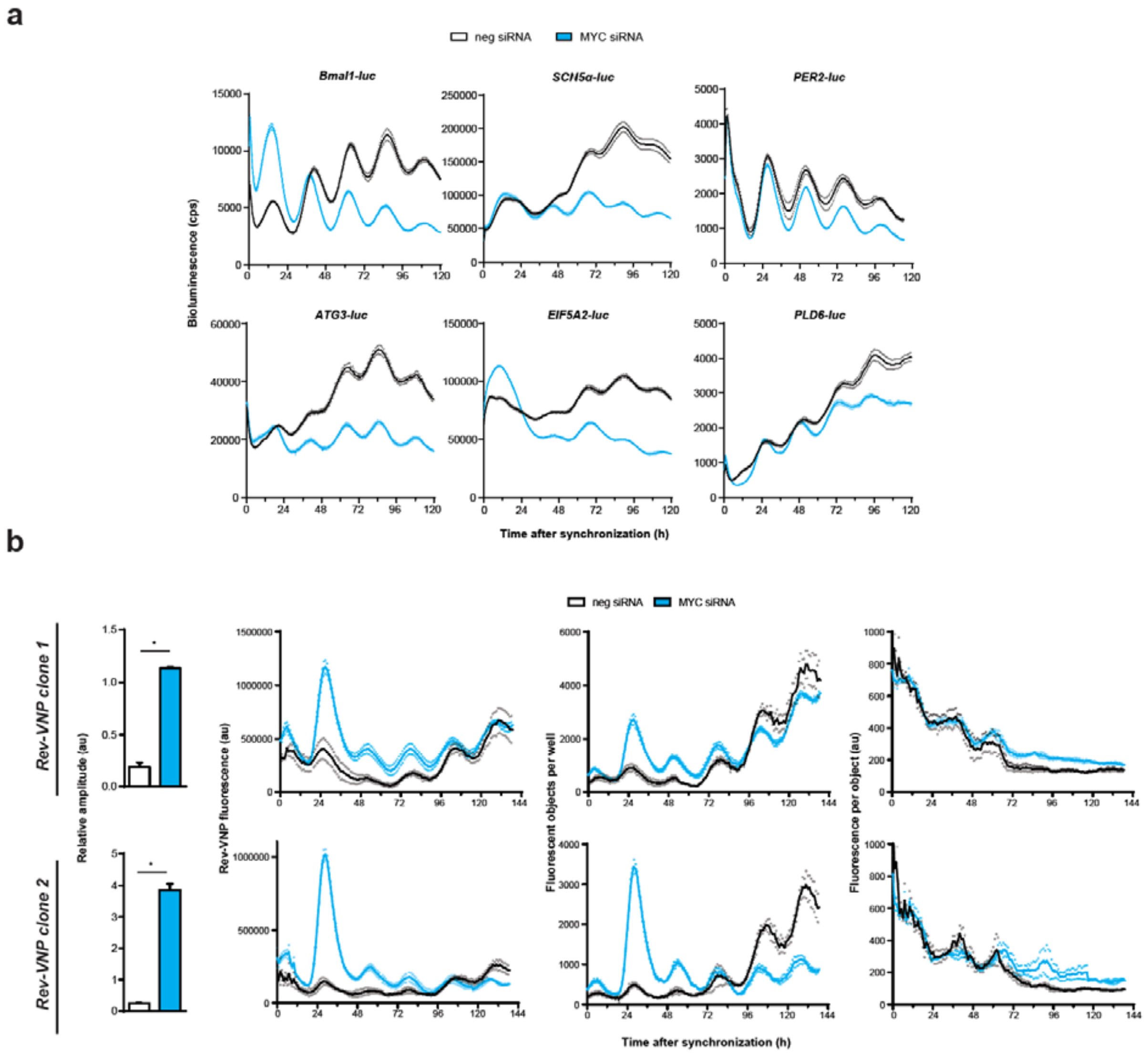

Supplementary Figure 5. Knockdown of MYC improves circadian rhythmicity of U2OS cells

(a) Bioluminescence traces from stable *Bmal1-luc*, *SCN5α-luc*, *PER2-luc, ATG3-luc, PLD6-luc* and *EIF5A2-luc* U2OS cell lines transfected with MYC siRNA and negative siRNA (n=3). **(b)** Relative amplitudes (ChronoStar software), total fluorescence, fluorescent objects, and intensity per objects traces quantified from U2OS *Rev-VNP* cells transfected with MYC siRNA and negative siRNA (n=3). Data are presented as mean ± SEM. * $P < 0.05$; Student's *t*-test.

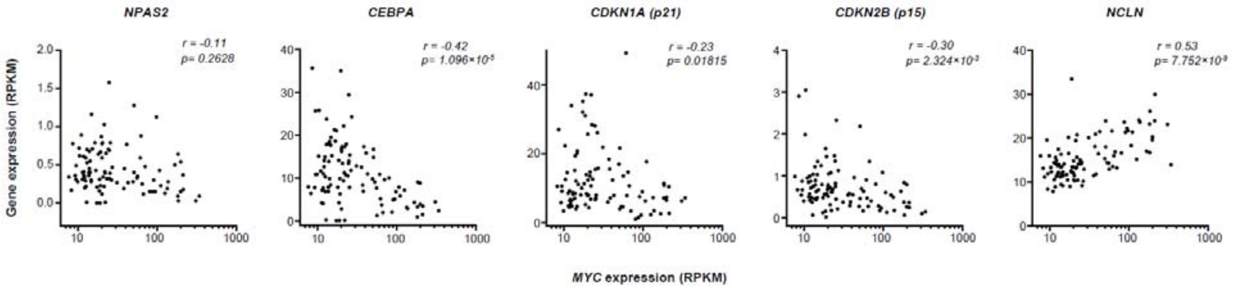

Supplementary Figure 6. Correlation of *MYC* expression with expression of known target genes in human lymphoma

Scatter plots of expression levels (RPKM) of *MYC* versus the indicated MYC-target genes in 102 human lymphoma samples of the ICGC MMML-Seq project[3].

a

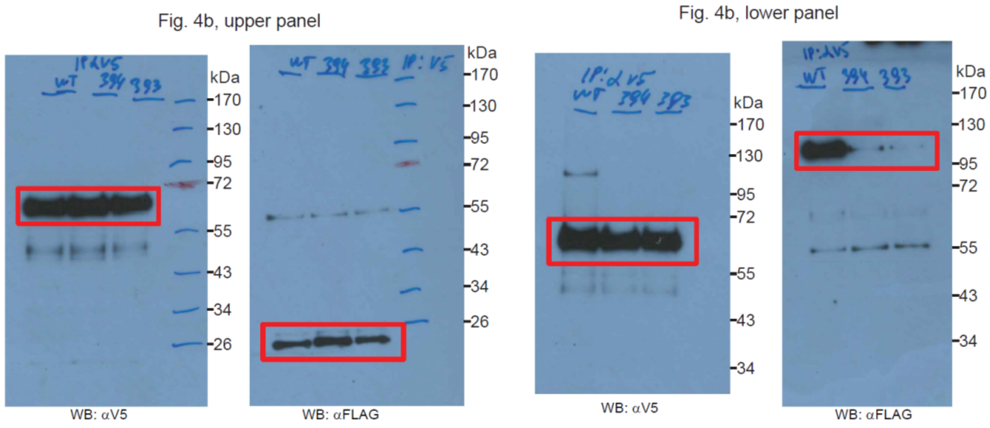
Fig. 4b, upper panel
Fig. 4b, lower panel
kDa
170
130
95
72
55
43
34
26
WB: αV5
WB: αFLAG
WB: αV5
WB: αFLAG

b

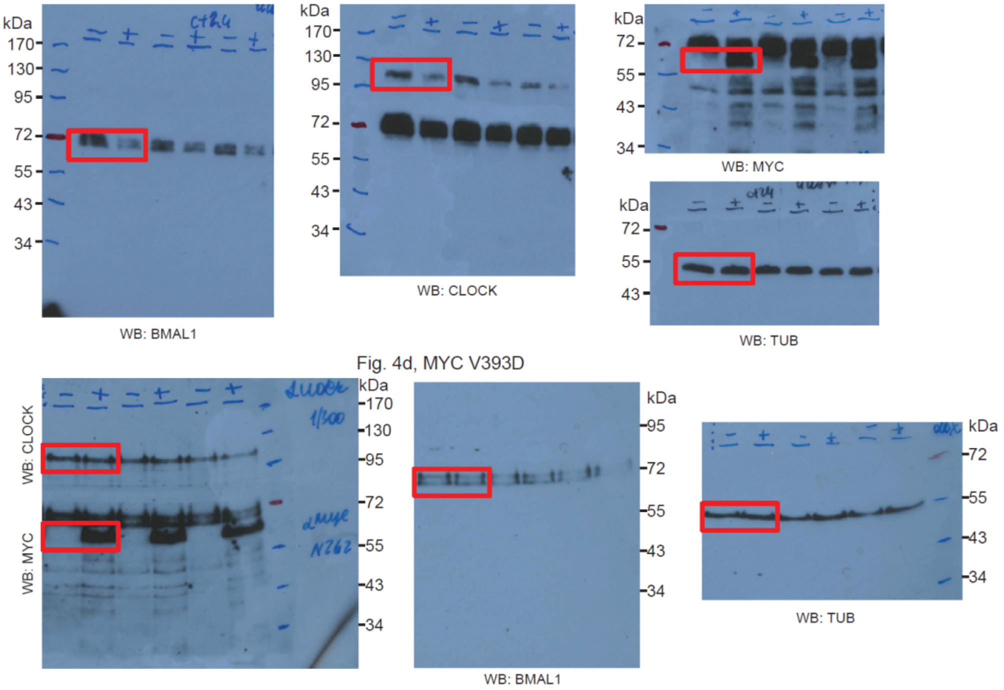
Fig. 4d, MYC WT
kDa
170
130
95
72
55
43
34
WB: BMAL1
WB: CLOCK
WB: MYC
WB: TUB
Fig. 4d, MYC V393D
WB: CLOCK
WB: MYC
WB: BMAL1
WB: TUB

c

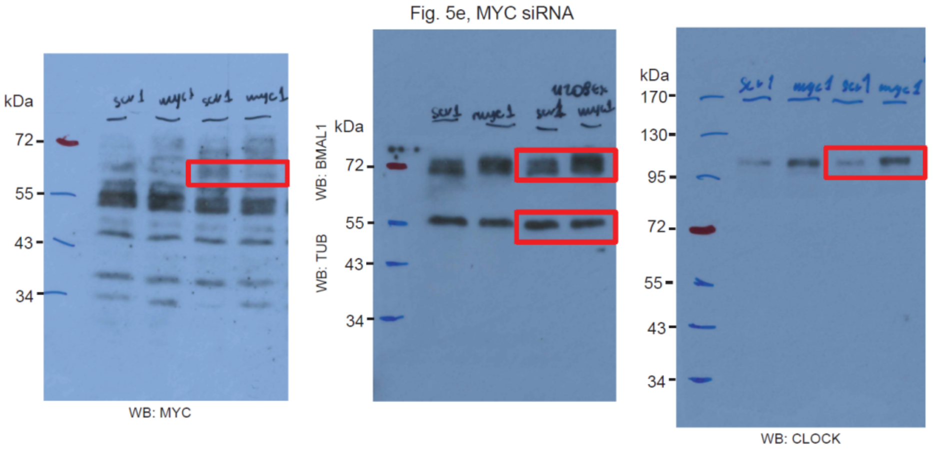
Fig. 5e, MYC siRNA
kDa
170
130
95
72
55
43
34
WB: MYC
WB: BMAL1
WB: TUB
WB: CLOCK

Supplementary Figure 7. Full scans of Western blots shown in main figures

Cropped areas are marked by red color.

Supplementary Table 1. Primer sequences and siRNAs

qPCR primers for gene expression analysis		
**name**	**sequence**	**source**
hGAPDH_F	TGCACCACCAACTGCTTAGC	Zhang, E.E. *et al.* (2009) A genome-wide RNAi screen for modifiers of the circadian clock in human cells. *Cell*
hGAPDH_R	ACAGTCTTCTGGGTGGCAGTG	
hBMAL1_F	GCCCATTGAACATCACGAGTAC	
hBMAL1_R	CCTGAGCCTGGCCTGATAGTAG	
hCLOCK_F	GGCACCACCCATAATAGGGTA	
hCLOCK_R	TGTTGCCCCTTAGTCAGGAAC	
hPER2_F	AGTTGGCCTGCAAGAACCAG	
hPER2_R	ACTCGCATTTCCTCTTCAGGG	
hREV-ERBa_F	TGGACTCCAACAACAACACAG	
hREV-ERBa_R	GTGGGAAGTAGGTGGGACAG	
hMYC_F	CACCAGCAGCGACTCTGA	Walz, S. et al. (2014) Activation and repression by oncogenic MYC shape tumour-specific gene expression profiles. *Nature*
hMYC_R	GATCCAGACTCTGACCTTTTGC	
hMIZ1_F	TGAAGATCCACATCGCTGACG	
hMIZ1_R	GGTCTGCAAACTGTCGCTG	
hNPAS2_F	CGTGTTGGAAAAGGTCATCGG	**PrimerBank ID 48928051c1**
hNPAS2_R	TCCAGTCTTGCTGAATGTCAC	
hREV-ERBβ_F	TCATGCTTGCGAAGGCTGTAA	**PrimerBank ID224177492c2**
hREV-ERBβ_R	CGCTTAGGAATACGACCAAACC	
FLAG:hCLOCK_F	TGGACTACAAAGACGATGACG	**This work**
FLAG:hCLOCK_R	AGCATTACCAGGAAGCATGG	

qPCR primers for ChIP		
**name**	**sequence**	**source**
neg region chr11_F	TTTTCTCACATTGCCCCTGT	Walz, S. *et al.* (2014) Activation and repression by oncogenic MYC shape tumour-specific gene expression profiles. *Nature*
neg region chr11_R	TCAATGCTGTACCAGGCAAA	
hPER2_Ebox_F	GAGGGCGTAGTGAATGGAAG	**This work**
hPER2_Ebox_R	GCCCGTCGCTCTTTTTACAT	
hNCL_Ebox_F	GGGACTCGACTCCTGACG	
hNCL_Ebox_R	ACTCCGACTAGGGCCGATAC	
hREV-ERBa_Ebox_F	TCTCGATTGACGGGAAGC	
hREV-ERBa_Ebox_R	CTCACGTCCCTGCTCCAC	
hNPAS2_MIZ1bs_F	CGCCGCTCATTGAGAAAT	
hNPAS2_MIZ1bs_R	ACTGTCACCCCCTCCACAC	
hCLOCK_MIZ1bs_F	GCGGCTATTAGCGTCTGACT	
hCLOCK_MIZ1bs_R	TTCACTGGCGGAAAGAAATC	

hBMAL1_ench_MIZ1bs_F	TCCTGACTGCCACAGATCAA	
hBMAL1_ench_MIZ1bs_R	TCCCTCCTGTTCCAAGTGAG	
hBMAL1_TSS_MIZ1bs_F	GGATTGGTCGGAAAGTAGGTT	
hBMAL1_TSS_MIZ1bs_R	CGGGTAAACAGGCACCTC	

siRNA sequences		
**name**	**sequence**	**source**
negative siRNA	N/A	Silencer Select Negative Control No. 1 siRNA, cat # 4390843, Ambion
hMYC	N/A	sc-29226, SantaCruz
hREV-ERBα	CAGCAGAACAUCCAGUACAAAdtdt	Baggs, JE. *et al.* (2009) Network Features of the Mammalian Circadian Clock *PLOS Biology.*
hMIZ1#1	AGUUCAACCAGGUAGGGAAdtdt	Kaur, M. *et al.* (2013) MYC acts via the PTEN tumor suppressor to elicit autoregulation and genome-wide gene repression by activation of the Ezh2 methyltransferase. *Cancer Research.*
hMIZ1#2	GGUGGACGGUGUUCACUUUdtdt	Walz, S. *et al.* (2014) Activation and repression by oncogenic MYC shape tumour-specific gene expression profiles. *Nature*

References

1 Hoffmann, J. *et al.* Non-circadian expression masking clock-driven weak transcription rhythms in U2OS cells. *PLoS One* **9**, e102238 (2014).

2 Walz, S. *et al.* Activation and repression by oncogenic MYC shape tumour-specific gene expression profiles. *Nature* **511**, 483-487 (2014).

3 Richter, J. *et al.* Recurrent mutation of the ID3 gene in Burkitt lymphoma identified by integrated genome, exome and transcriptome sequencing. *Nature genetics* **44**, 1316-1320 (2012).
